# Computation of the gradient-induced electric field noise in 12-lead ECG traces during rapid MRI sequences

**DOI:** 10.1186/1532-429X-16-S1-P151

**Published:** 2014-01-16

**Authors:** Shelley H Zhang, Zion T Tse, Wei Wang, Raymond Y Kwong, Charles L Dumoulin, Ehud J Schmidt

**Affiliations:** 1 Brigham and Women's Hospital, Boston, Massachusetts, USA; 2 Engineering, The University of Georgia, Athens, Georgia, USA; 3 Radiology, Cincinnati Children's Hospital Medical Center, Cincinnati, Ohio, USA

## Background

Successful physiological monitoring using a 12-lead ECG during MR imaging is essential for safe conduction of cardiovascular interventions within a MR scanner. However, ECG artifacts induced by magnetic field gradients severely affect the signal quality and fidelity. Previously, the gradient-induced artifacts were reduced by blocking ECG transmissions during all gradient ramps [1], which has been shown feasible while the method is not suitable for short-TR sequences. Theoretical and experimental studies have shown a linear relationship between electric fields and the temporal derivatives of the magnetic field gradients [2,3]. We propose an algorithm to restore the true ECG signal by subtracting system response functions, based on the MR gradient signals, from ECG signals distorted by gradient interference.

## Methods

Data Acquisition: An MRI-conditional 12-lead ECG system [1] was used to acquire data on two healthy volunteers inside a 3T MRI. Outside the MRI room, high-fidelity ECG traces, along with the x, y and z gradient waveforms were digitally recorded simultaneously at 62kHz. Balanced SSFP sequences with various slice orientations (axial, coronal, sagittal and oblique) were acquired. Data Analysis: The gradient-induced ECG noise was computed as the difference between aligned ECG traces with and without MR sequence running. The noise voltage (Vni) at each electrode (i) was modeled as a linear combination of gradient derivatives and system factors, Vni = αi•dGx/dt+βi•dGy/dt+γi•dGz/dt+Ci, where αi, βi, γi and Ci are position-dependent. These parameters were then used to reconstruct the noise, for comparison with the measured ECG noise, and to further derive the restored ECG.

## Results

**Figure 1:**
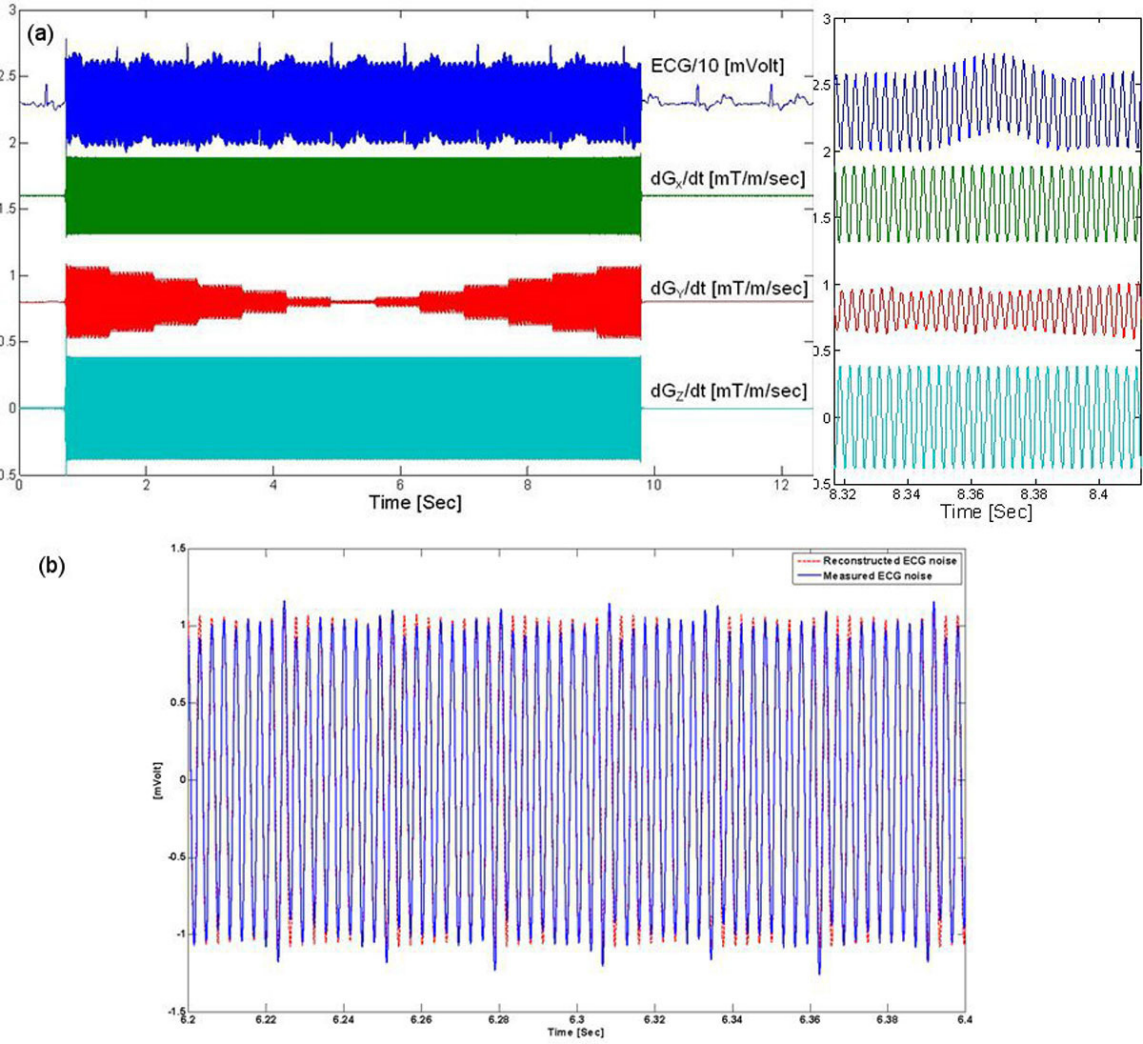
**(a): One representative ECG trace (V5) and gradient waveforms derivatives from the X, Y and Z axes during a transverse (Z, or Superior-Inferior, slice encode) SSFP acquisition, with frequency encoding along × (Right-Left) and phase encoding along Y (Anterior-Posterior)**. A time domain magnification (right), shows the gradient derivative signals (only 0 to 500 Hz are shown). (b): Plots of measured ECG noise (solid blue) and computed ECG noise vector (dashed red) based on the fitted parameters. There is an 18% difference between the reconstructed and measured ECG noise.

**Figure 2:**
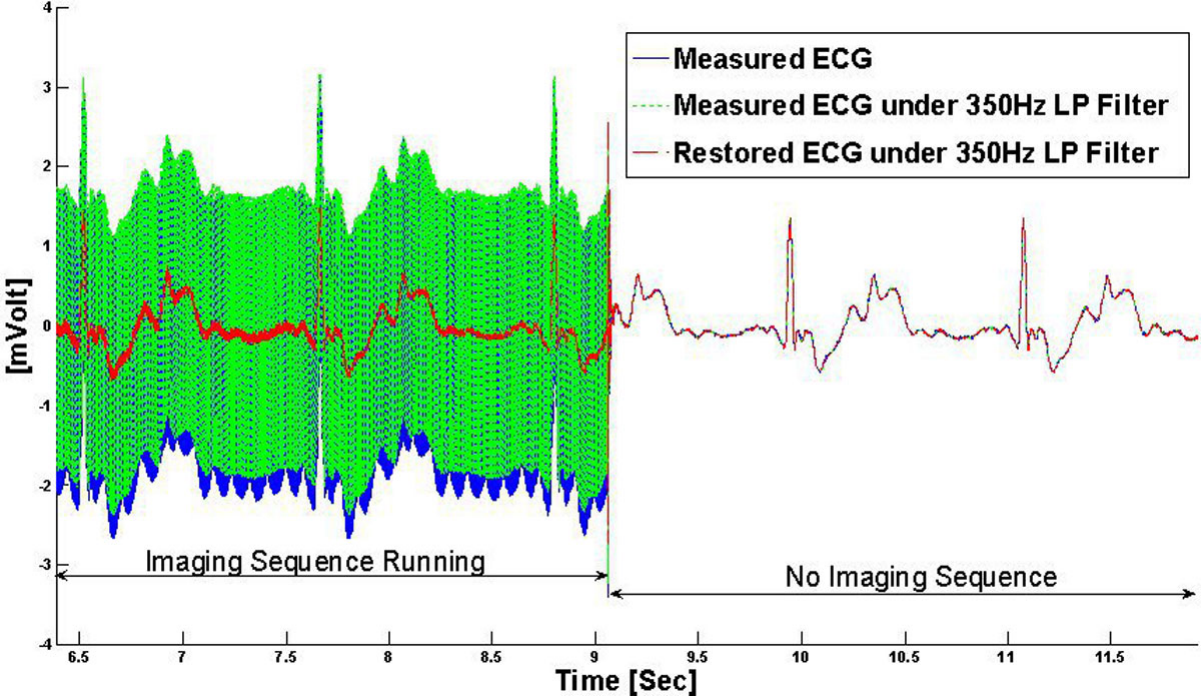
**During Imaging, the restored ECG (red line) signal preserves the same signal shape as the ECG has in the absence of imaging (no gradient switching), while low-pass (LP) filtering (green dashed line) fails to clean the gradient-induced artifacts**.

**Figure 3:**
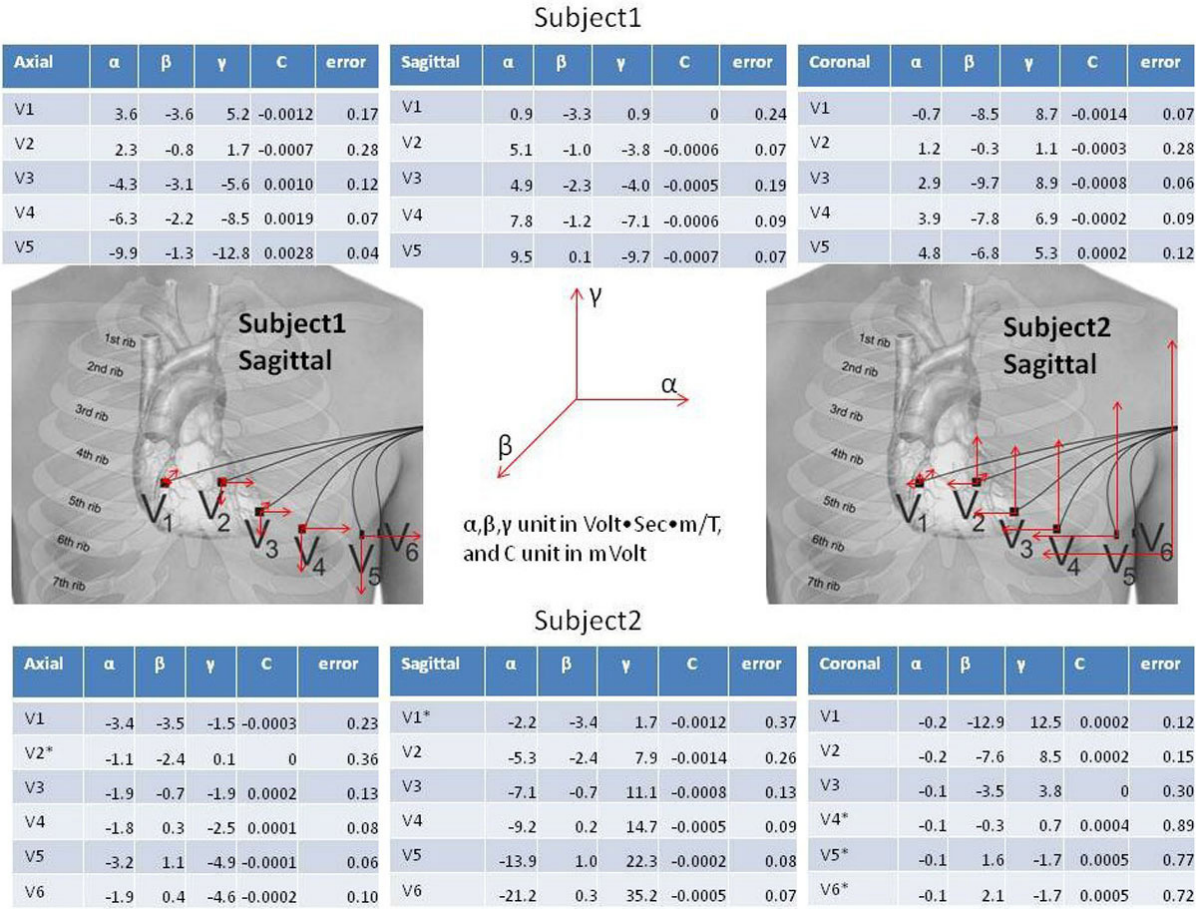
**The fitted parameters for three SSFP acquisition orientations are listed for Subject 1 (top) and subject 2 (bottom)**. The 3D vector plots in the center illustrate graphically sagittal acquisitions in both subjects utilizing phase-encoding along Y (Anterior-Posterior) for the precordial electrodes V1-V6. A gradually increasing influence of the magnetic gradient fields on the ECG noise was observed from V1 to V6.

The recorded ECG traces and low-pass filtered gradient derivatives are displayed in Figure 1a. The computed noise vector (Vni) and the measured noise (Figure 1b) had differences of 21% ± 20% in normalized Euclidean distance. The restored ECG signal was comparable to the clean ECG segments (Figure 2), providing higher signal quality and fidelity relative to low-frequency filtering of the ECG signal. Vectorial display of the fitted parameters (Figure 3) demonstrated systematic changes across the precordial leads, and varied in magnitude between subjects.

## Conclusions

The gradient-derivative model closely fit the measured ECG noise, possibly allowing for efficient gradient-noise removal utilizing rapid calibration scans, combined with hardware blocking of extremely high noise intervals.

## Funding

NIH U41-RR019703, R03 EB013873-01A1, and SBIR-1R43HL110427-01; AHA10SDG261039.
